# Assessment of an Innovative Mobile Dentistry eHygiene Model Amid the COVID-19 Pandemic in the National Dental Practice–Based Research Network: Protocol for Design, Implementation, and Usability Testing

**DOI:** 10.2196/32345

**Published:** 2021-10-26

**Authors:** Jin Xiao, Cyril Meyerowitz, Patricia Ragusa, Kimberly Funkhouser, Tamara R Lischka, Luis Alberto Mendez Chagoya, Nisreen Al Jallad, Tong Tong Wu, Kevin Fiscella, Eden Ivie, Michelle Strange, Jamie Collins, Dorota T Kopycka-Kedzierawski, National Dental Practice-Based Research Network Collaborative Group

**Affiliations:** 1 Eastman Institute for Oral Health University of Rochester Medical Center Rochester, NY United States; 2 Kaiser Permanente Center for Health Research Portland, OR United States; 3 Department of Biostatistics and Computational Biology University of Rochester Medical Center Rochester, NY United States; 4 Department of Family Medicine University of Rochester Medical Center Rochester, NY United States; 5 Mouthwatch LLC Metuchen, NJ United States

**Keywords:** teledentistry, mDentistry, oral diseases, virtual visit, intraoral camera, pandemic response, COVID-19, mHealth

## Abstract

**Background:**

Amid COVID-19, and other possible future infectious disease pandemics, dentistry needs to consider modified dental examination regimens that render quality care, are cost effective, and ensure the safety of patients and dental health care personnel (DHCP). Traditional dental examinations, which number more than 300 million per year in the United States, rely on person-to-person tactile examinations, pose challenges to infection control, and consume large quantities of advanced-level personal protective equipment (PPE). Therefore, our long-term goal is to develop an innovative mobile dentistry (mDent) model that takes these issues into account. This model supplements the traditional dental practice with virtual visits, supported by mobile devices such as mobile telephones, tablets, and wireless infrastructure. The mDent model leverages the advantages of digital mobile health (mHealth) tools such as intraoral cameras to deliver virtual oral examinations, treatment planning, and interactive oral health management, on a broad population basis. Conversion of the traditional dental examinations to mDent virtual examinations builds upon (1) the reliability of teledentistry, which uses intraoral photos and live videos to make diagnostic decisions, and (2) rapid advancement in mHealth tool utilization.

**Objective:**

In this pilot project, we designed a 2-stage implementation study to assess 2 critical components of the mDent model: virtual hygiene examination (eHygiene) and patient self-taken intraoral photos (SELFIE). Our specific aims are to (1) assess the acceptance and barriers of mDent eHygiene among patients and DHCP, (2) assess the economic impact of mDent eHygiene, and (3) assess the patient’s capability to generate intraoral photos using mHealth tools (exploratory aim, SELFIE).

**Methods:**

This study will access the rich resources of the National Dental Practice-Based Research Network to recruit 12 dentists, 12 hygienists, and 144 patients from 12 practices. For aims 1 and 2, we will use role-specific questionnaires to collect quantitative data on eHygiene acceptance and economic impact. The questionnaire components include participant characteristics, the System Usability Scale, a dentist-patient communication scale, practice operation cost, and patient opportunity cost. We will further conduct a series of iterative qualitative research activities using individual interviews to further elicit feedback and suggestion for changes to the mDent eHygiene model. For aim 3, we will use mixed methods (quantitative and qualitative) to assess the patient’s capability of taking intraoral photos, by analyzing obtained photos and recorded videos.

**Results:**

The study is supported by the US National Institute of Dental and Craniofacial Research. This study received “single” institutional review board approval in August 2021. Data collection and analysis are expected to conclude by December 2021 and March 2022, respectively.

**Conclusions:**

The study results will inform the logistics of conducting virtual dental examinations and empowering patients with mHealth tools, providing better safety and preserving PPE amid the COVID-19 and possible future pandemics.

**International Registered Report Identifier (IRRID):**

PRR1-10.2196/32345

## Introduction

### Urgent Need to Transform the Dental Visit Format During Infectious Disease Outbreaks

Amid the COVID-19 pandemic, dental health care personnel (DHCP) are at great risk of contracting the SARS-CoV-2 virus due to their close physical proximity to their patients, as well as the enhanced potential for transmission of airborne viruses in the dental setting [1]. When delivering dental services, DHCP consume increased amounts and enhanced levels of personal protective equipment (PPE), equivalent to the ones used by other health care providers who provide care to COVID-19–positive patients. This PPE includes N95 respirators, goggles, gowns, head covers, and face shields. Although dentistry has practiced for years utilizing person-to-person visual-tactile examinations, now, more than ever, utilizing a wide variety of new technologies and approaches to deliver virtual dental services would have significant utility.

Aggressively converting traditional dental examinations (eg, comprehensive, limited, and hygiene recall examinations) to virtual examinations could significantly reduce the exposure risk for patients and DHCP and preserve a large volume of PPE essential to the medical and dental communities. According to the American Dental Association, as of 2019, 200,419 dentists were practicing dentistry in the United States, with 158,331 (79%) general dentists (GD) providing a range of examination visits on a daily basis [2]. GDs in the United States are conducting 564 million patient visits per year (an average of 3566 patient visits per GD) [3]. Importantly, 316 million of these 564 million patient visits (56%) are examination visits. These visits comprise 67 million (21%) new patient examinations, 40 million (13%) limited examinations, and 209 million (66%) hygiene recall examinations, which are often not linked to definite treatment delivery at the same visit. If these 316 million examinations were all (or partially) converted to virtual visits, serving as remote triage of patients’ needs, 316 million person-to-person contacts would be avoided, preserving at least 1 billion pieces of PPE per year.

**Figure 1:**
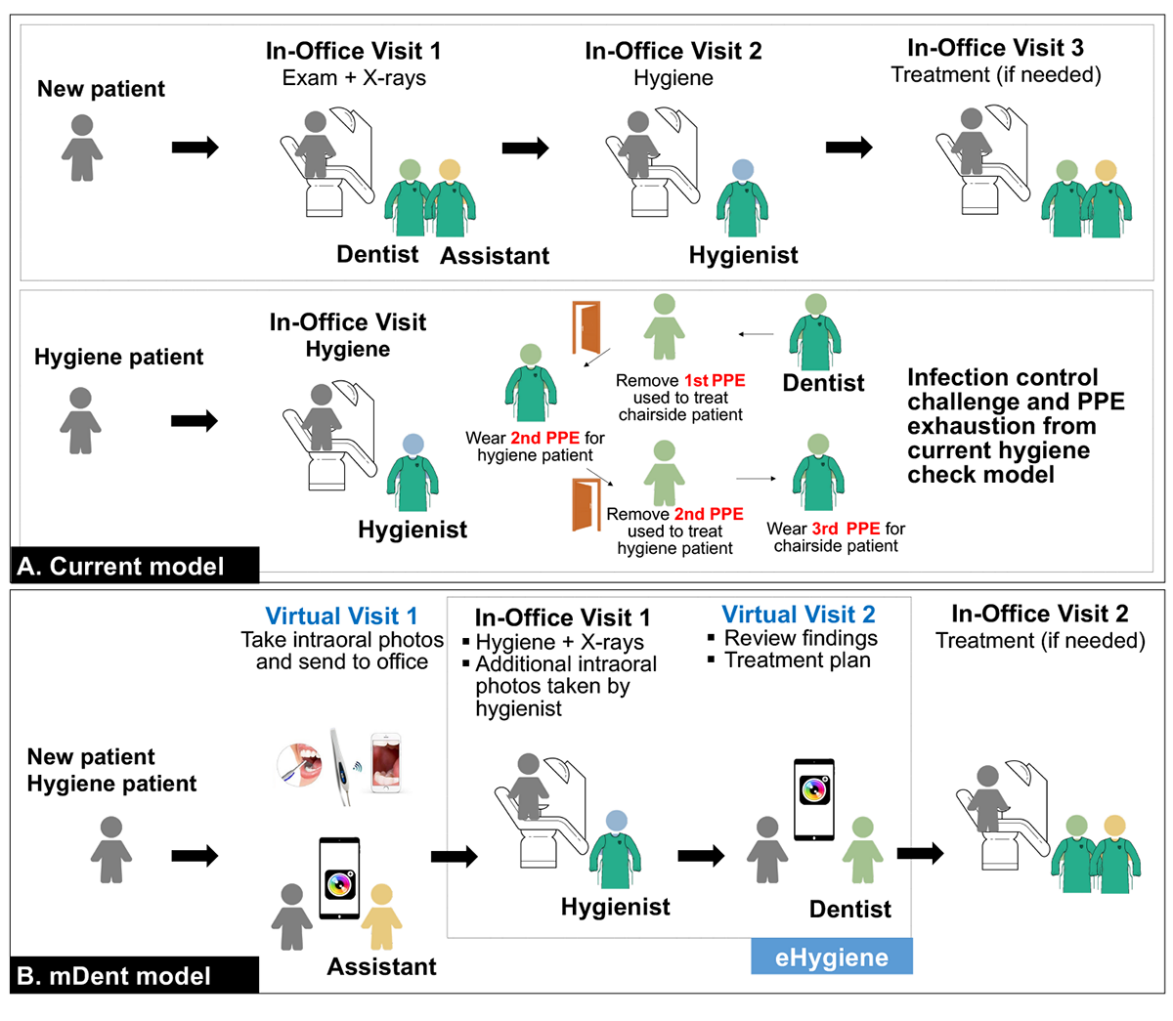
Current and proposed mobile dentistry (mDent) model for dental examinations. PPE: personal protective equipment.

In the current dental examination model (Figure 1), patients often need to have one examination visit and one hygiene visit before they get to a definite treatment visit. A dentist hygiene check visit usually includes x-rays taken by the dental hygienist and an examination completed by the dentist [4]. In a regular dental office, the dentists usually conduct hygiene examinations in between treating their chairside patients [4]. In the current COVID-19 environment and for the foreseeable future, the dentist needs to change PPE between seeing his or her chairside patient and the hygiene patient and then change to another PPE when completing the hygiene check and returning to the original chairside patient. A single hygiene check examination consumes 2 sets of PPE for the dentist alone and increases the challenge of infection control due to frequent switching of PPE and the dentist running between dental operatories. Moreover, with added time from changing PPE, extended waiting can add more frustration to that already reported by patients and dental hygienists while waiting for dentists during traditional hygiene examinations [4]. Dentistry needs changes to the dental examination regimens, especially hygiene examinations, to render quality care and ensure the safety of patients and the DHCP amid the COVID-19 outbreak.

**Rationale for Developing the mDent eHygiene Model in a Digital Era**

Our long-term goal is to develop an innovative mobile dentistry [5] (mDent) model in this digital era (illustrated in Figure 1). The mDent model refers to the practice of dentistry supported by mobile devices such as mobile telephones, tablets, personal digital assistants, and a wireless infrastructure. The mDent model combines virtual dental visits with the use of digital mobile health (mHealth) tools, such as intraoral cameras, to complete oral health screening, treatment planning, virtual hygiene examinations, and interactive oral health education, on a broad population basis. In the mDent model, before patients arrive at the dental office, they would have a virtual visit with dental office personnel to take a series of intraoral photos at home. Capable patients could do this independently by watching a photo-taking tutorial video, minimizing DHCP instruction time during a virtual visit. With the intraoral photos, the DHCP would have a preliminary idea of the patient’s oral health. The second visit would be an in-office hygiene visit conducted by the dental hygienist to complete intraoral x-ray records, a soft and hard tissue examination, and additional intraoral photos, if needed. Patients will then have a virtual dental visit, scheduled at a convenient time with the dentist, to review the findings and treatment plans before they proceed with an in-office visit to confirm the examinations and receive a definite dental treatment plan and dental treatment, as appropriate.

This mDent model will fully engage relevant stakeholders (patients, dental hygienists, and dentists) to conduct interactive oral health practices. The mDent model will also utilize a patient-driven mobile device to increase the accessibility of dental care. Moreover, in the era of COVID-19 risk, this remote virtual dental service model will lead to a well-planned dental service, better infection control, and reduced PPE consumption. As this eHygiene implementation study is a hypothesis-generating pilot study, our immediate objective is to assess the following 3 aims in the National Dental Practice-Based Research Network [6]: (1) Aim 1 is to assess the acceptance and barriers of mDent eHygiene among patients and DHCP, (2) aim 2 is to assess the economic impact of mDent eHygiene, (3) and aim 3 is to assess patients’ capability for generating intraoral photos using mHealth tools (SELFIE).

## Methods

### Overall Study Design

**Figure 2:**
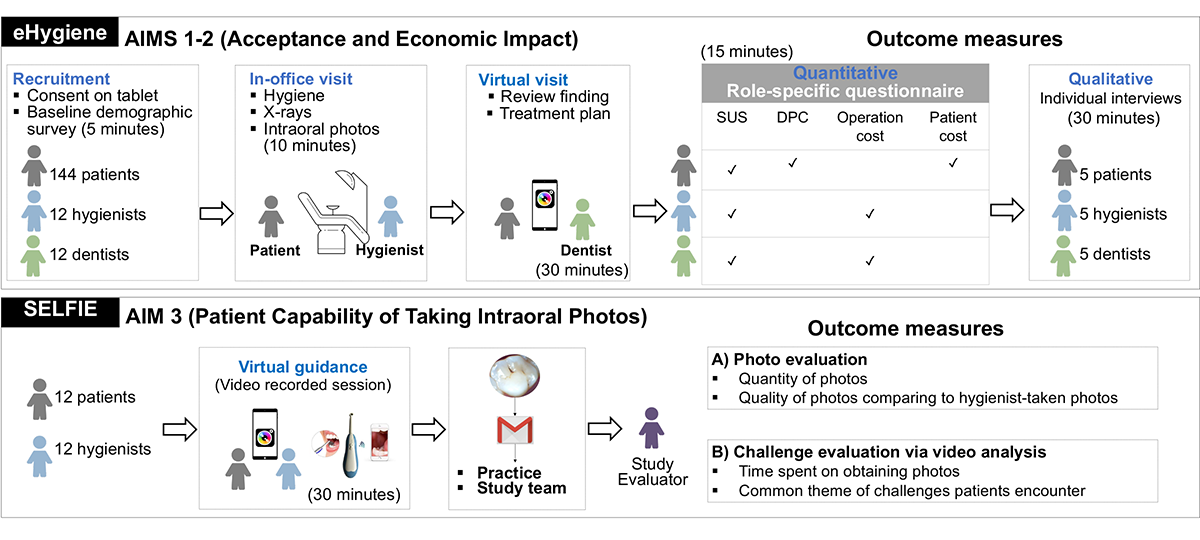
Specific aims, study design, and outcome measures. DPC: dentist-patient communiation; SUS: System Usability Scale.

This study will use a 2-stage implementation study to assess the acceptance of 2 components (eHygiene and SELFIE) of the mDent eHygiene model among patients and DHCP (dentists and dental hygienists). The components are illustrated in Figure 2.

This mDent eHygiene study will use mixed methods (quantitative and qualitative) to collect outcome measures and conduct data analysis. For details, see Table 1 and the following subsections.

**Table 1:** Mobile dentistry (mDent) model eHygiene objectives and outcome measures.

Aims	Outcome measurements	Brief description and justification of the outcome measures
Aim 1: assess the acceptance of and barriers to mDent eHygiene among patients and DHCP^a^	System Usability Scale (SUS); dentist-patient communication (DPC); theme of acceptance and barriers analyzed from individual qualitative interviews with the DHCP	The SUS instrument will be used to assess the acceptance of the mDent eHygiene approach. The SUS instrument [7-9] is widely adopted in business and technology industries and mHealth^b^ fields to measure and quantify the perception of product and service usability. The DPC component will be used to assesses how well the patients understand the planned treatment and the quality of the communication between the patients and dentists using eHygiene. We will use a modified questionnaire from a validated DPC questionnaire [10]. Qualitative analysis will be conducted for data from 15 individuals (5 patients, 5 dentists, 5 dental hygienists) via 30-minute virtual individual interviews. The questions during the interview will address the feedback, perceived challenges, and suggestions for improvement for the mDent eHygiene model.
Aim 2: assess the economic impact of mDent eHygiene	PPE^c^ consumption and estimated cost and eHygiene chair time per patient; eHygiene virtual visit time per patient; DHCP (dentist and dental hygienist) personnel cost related to eHygiene	Studies from other groups have shown improved cost-effectiveness using virtual dental visits [11,12]. Now, facing the PPE shortage amid the COVID-19 outbreak, the economic benefits of mDent eHygiene are promising. The magnitude, however, must be carefully evaluated, which will be assessed using the outcome measured listed in this objective via role-specific baseline and post-eHygiene questionnaires.
Aim 3: assess patients’ capability for generating intraoral photos using mHealth tools (SELFIE)	Quantity and quality of intraoral photos taken by patients, assessed by 1 dentist in the study team who will be trained for photo assessment; themes of challenges encountered by patients while taking intraoral photos themselves, by analyzing the video recordings from the SELFIE session	This objective will provide preliminary data on patient engagement with using mHealth tools, which is essential to empowering patients in the complete mDent model.

^a^DHCP: dental health care personnel.

^b^mHealth: mobile health.

^c^PPE: personal protective equipment.

### Participants and Recruitment

The mDent eHygiene study will be conducted in the Northeastern node of the National Dental Practice-Based Research Network in the United States. This mDent eHygiene study will enroll 144 patients and 24 DHCPs from 12 practices. Each practice will enroll 12 patients, 1 dentist, and 1 dental hygienist. All 144 patients and 24 DHCPs will conduct the eHygiene session (1st stage) of the study. Among enrolled patients and DHCPs, 5 patients, 5 dentists, and 5 dental hygienists will be invited for a 30-minute recorded telephone interview for a qualitative session of the eHygiene study. In addition, 12 patients will be invited to conduct a SELFIE session to evaluate their capability for taking intraoral photos by themselves, with guidance from the dental hygienist.

### Intraoral Photo Taken by a Dental Hygienist in an eHygiene Session

**Figure 3:**
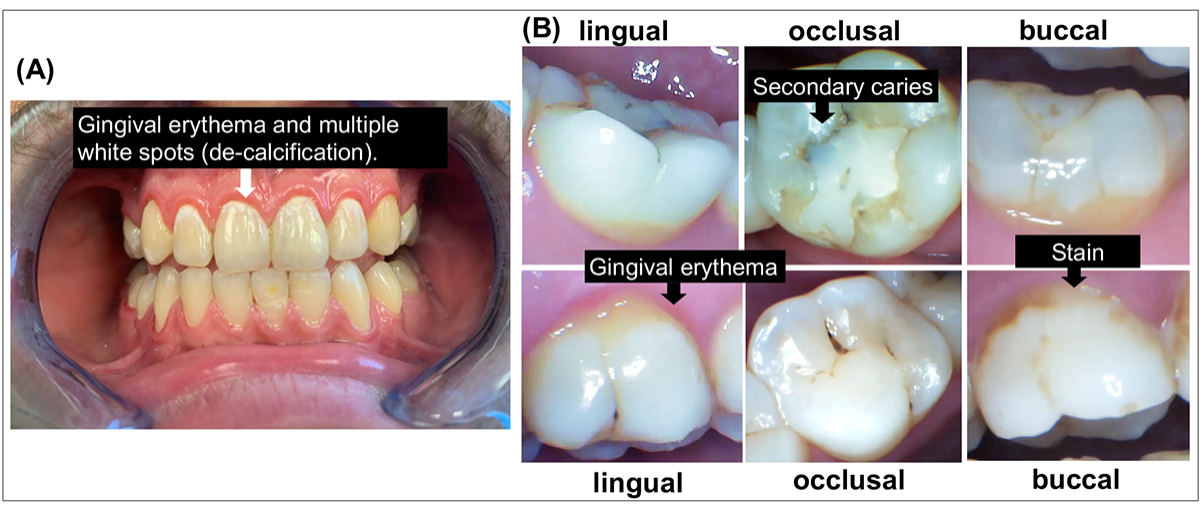
Patient self-taken intraoral photos: (A) front view taken on an iPhone X, on which gingival erythema and multiple white spots (de-calcification) are seen; (B) posterior photos taken with a Mouthwatch intraoral camera, where the upper panel includes photos of the lower left first molar and lower panel photos of the upper right first molar.

The dental hygienist will complete a routine hygiene visit, update x-rays, and perform a hard and soft tissue examination as part of routine clinical care. Intraoral photos of patients will be taken for research purposes following a recommended template (See Multimedia Appendix 1). Intraoral photos will be stored in Mouthwatch Teledent—a cloud-based platform for conducting Teledent visits. A series of intraoral photos will be taken by a tablet and an intraoral camera connected to a tablet. Examples of intraoral photos are shown in Figure 3.

### Patient-Dentist Virtual Visit in the eHygiene Session

The dentist will then conduct a virtual visit with the patient lasting approximately 30 minutes at a later and suitable time within 14 days of the eHygiene intraoral photo visit, to review eHygiene findings and treatment plans.

### SELFIE Session

The self-taking of intraoral photos (SELFIE session) will be piloted by 12 (1 from each practice) of the 144 study patients who complete the eHygiene session. The intraoral camera and instructional video will be given to the patient when the patient leaves the hygiene visit. During a virtual visit with the dental hygienist, the patient will use an intraoral camera while being supervised by the dental hygienist to take a series of intraoral photos of the front and posterior teeth. This virtual visit session will be recorded.

Patients will be encouraged to think-aloud [13] about their feelings and difficulties encountered while taking photos. The think-aloud process asks users to verbalize their thoughts as they complete various tasks, allowing investigators to gain insight on participants’ thought processes in relation to the technology products.

### Study Questionnaires

Each participating patient, dentist, and dental hygienist will complete baseline and post-eHygiene role-specific questionnaires (see Multimedia Appendix 2 for the dentist questionnaire, Multimedia Appendix 3 for the hygienist questionnaire, and Multimedia Appendix 4 for the patient questionnaire) as per the study schedule. The questionnaires include the System Usability Scale (SUS), dentist-patient communication (DPC) scale, office operation, and other items detailed in Table 1.

### Qualitative Interviews

After receiving the SUS scores from all patients and DHCPs, the study team will randomly select 15 individuals (5 patients, 5 dentists, 5 hygienists) for virtual individual interviews lasting approximately 30 minutes. These 15 individuals will include those who rated above and below the average SUS score. The questions asked during the interview will address feedback and recommendations, perceived challenges, and suggestions for improvement of the mDent eHygiene model. The interviews will be standardized using an interview guide (see Multimedia Appendix 5), and interviews will be audio-recorded.

### Statistical Analysis

#### Sample Size Considerations

The sample size calculation for the primary outcome (eHygiene—SUS) was based on the primary outcome of the SUS score from patients. Various studies [7,14,15] have used the SUS scale to assess the usability of a medical service or mHealth tool and reported mean SUS scores of 47.5-81.2 (SD 9.9-21.1). Since the patients in the eHygiene study are clustered by practice, we used a cluster randomized design calculation for the sample size calculation. Assuming the difference in the SUS score between the patient-evaluated eHygiene model and other published mHealth tools has a mean of 8 and an SD of 10, a sample size of 72 patients from 12 practices (6 per practice) will achieve 90% power, at an alpha of .05. Considering the potential dropout rate, a sample size of 144 patients will satisfy the statistical power of the primary outcome.

Dentists and dental hygienists will complete the SUS for each patient when the dentist and hygienist complete an eHygiene visit, which means each dentist and hygienist will evaluate the eHygiene model 12 times. Assuming a mean difference of 30 (SD 20) between the first and last patients evaluated by the dentists and hygienists, using a paired *t* test, a sample of 7 dentists or hygienists will achieve 90% power, at an alpha of .05. Considering potential dropouts, recruiting 12 dentists and 12 dental hygienists will achieve satisfactory statistical power for this aim.

Using a cluster randomized design to calculate the sample size for the primary outcome of DPC and assuming a mean difference in the patient-evaluated DPC score between the current hygiene model and the eHygiene model of 8 (SD 10), a sample size of 48 patients from 12 practices (4 per practice) will achieve 85% power, at an alpha of .05. A sample size of 144 patients will satisfy the statistical power of the DPC outcome, while considering potential dropouts.

For the sample size calculation of the tertiary outcome (SELFIE), we expect to reach data saturation [16] (no new themes are identified) after conducting 12 individual tests. The sample size was determined based on previously published studies, where between 6 and 11 usability tests were conducted to assess technology products, for instance, smartphone app usability [17,18].

#### Analyses for Aim 1: eHygiene

We will calculate SUS scores for the eHygiene model (post-eHygiene SUS) as rated by patients, dentists, and dental hygienists. The SUS score from the patients and DHCPs between practices will be compared. A linear mixed effects model will be used to examine factors that influence the SUS score as perceived by patients, including patient factors (demographic, socioeconomic, education, profession, and experience with using a digital device and mHealth tools) and DHCP factors (demographic and dental practice experience), while considering the clustering effects within practices and providers. The eHygiene SUS score as rated by dentists and dental hygienists from treating the first patient through the last study patient will be compared to assess whether the DHCP-determined SUS score is associated with a learning curve.

We will calculate the DPC score as rated by patients, which assesses how well the patients understand the planned treatment and the quality of the communication between the patients and dentists who participate in eHygiene. We will use a linear mixed effects model to examine factors that influence the DPC score perceived by patients, including patient factors (demographic, socioeconomic, education, profession, and experience with using a digital device and mHealth tools), DHCP factors (demographic and dental practicing experience), and time spent on the eHygiene visit, while controlling for the clustering effects within practices and providers. We will run separate models for patients and DHCP.

Regarding qualitative data, the interviews will be standardized using interview guides, audio-recorded, transcribed, coded, and analyzed for thematic content. The audio recordings will be transcribed by the Temi (San Francisco, CA) transcription service and further verified by 2 trained research personnel. Transcribed data will be analyzed using MAXQDA software (VERBI GmbH, Berlin, Germany). The data will be coded by 2 trained coders with predetermined open codes using a codebook with a description of the coding tree. Thematic content will be further analyzed using categorizing and contextualizing strategies to understand the factors associated with acceptance of and barriers to eHygiene among patients and DHCPs.

#### Analyses for Aim 2: Economic Impact

We will conduct analysis for the following parameters: (1) PPE consumption and estimated cost and comparison between eHygiene and traditional hygiene examination models for each practice; (2) eHygiene chair time per study patient, learning curve–related fluctuations in chair time per practice, and comparisons between practices; (3) eHygiene virtual visit time and comparison between practices; and (4) DHCP (dentist and dental hygienist) personnel cost related to eHygiene in-office and virtual visits, compared with traditional hygiene examination visits.

#### Analysis for Aim 3: SELFIE Intraoral Photos

Parameters to be evaluated include time spent on photo taking, number of photos, and readable photos by a dentist evaluator using a photo assessment from (Multimedia Appendix 6). Factors including patient demographic, education, and experience with using a digital device and mHealth tools that potentially relate to patient capability will be further assessed.

The “think-aloud” videos recorded during the SELFIE sessions will be reviewed by a trained study evaluator to analyze common themes of challenges patients encounter, using a SELFIE assessment form (Multimedia Appendix 7). The key tasks are connecting cameras with a tablet, locating the photo-taking module in the TeleDent software, using a cheek retractor, taking front-view and posterior teeth photos, and ensuring photos are stored in the TeleDent software. User performance for these key tasks will be ranked as critical (requiring assistance to proceed), severe (major delay and/or frustration), or cosmetic (minor) and annotated to the specific task. Based on rankings, the team will suggest changes to the instructional video and clinic procedures for future implementation.

## Results

The study has been peer-reviewed and funded by the US National Institute of Dental and Craniofacial Research. This study received single institutional review board approval from the University of Alabama at Birmingham (#300006506) and local context review from the University of Rochester (#6077). The eHygiene study is expected to launch in August 2021. Data collection and analysis are expected to conclude by December 2021 and March 2022, respectively.

## Discussion

### Study Innovation

This study is innovative in several ways. First, conducting virtual dental examinations (the mDent model) using intraoral photos and x-rays is novel and potentially transformative to dental practice. Using smartphones and mobile devices to take photos of the mouth and teeth and conduct oral disease screening has been recently reported [19-21]; however, the feasibility of engaging dental hygienists and patients to obtain intraoral images using an intraoral camera has not been assessed. Second, transforming the traditional one-on-one tactile dentist examination to an eHygiene visit requires a team effort from several stakeholders: the patient, dental hygienist, and dentist. This level of teamwork in dental offices is innovative. The team effort could lead to better DHCP-patient communication and a better understanding and compliance with this approach to dental treatment. Third, integrating the mHealth concept into dentistry to achieve population-wide oral health screening and monitoring is extremely innovative and offers a vehicle to promote patient-engaged oral health education and patient-driven early detection of oral disease. Fourth, the eHygiene model is a novel way of preserving PPE during the COVID-19 and other respiratory-transmissible disease outbreaks.

### Impact on Clinical Practice

Successful completion of this eHygiene pilot study will provide data on the acceptance and economic impact of virtual dental examinations during eHygiene visits, which will be a test vehicle for the future mDent model. The results will inform potential immediate modification of the dental service system to provide better safety and preserve PPE amid COVID-19 and other infectious disease outbreaks and beyond.

## Appendix

Multimedia Appendix 1 Intraoral photo taking template.

Multimedia Appendix 2 Dentist role-specific questionnaire.

Multimedia Appendix 3 Hygienist role-specific questionnaire.

Multimedia Appendix 4 Patient role-specific questionnaire.

Multimedia Appendix 5 Qualitative interview guide.

Multimedia Appendix 6 SELFIE session intraoral images assessment form.

Multimedia Appendix 7 SELFIE session video assessment form.

## Abbreviations

DHCP: dental health care personnel
DPC: dentist-patient communication
GD: general dentist
mDent: mobile dentistry
mHealth: mobile health
NIDCR: National Institute of Dental and Craniofacial Research
PPE: personal protective equipment
SUS: System Usability Scale

## References

[1] Gamio L (2020). The Workers Who Face the Greatest Coronavirus Risk. The New York Times.

[2] Workforce. American Dental Association.

[3] (2019). Average number of yearly patient visits per U.S. dentist from 1990 to 2018. Statista.

[4] Hurley J (2002). ‘Doctor, I need a hygiene check!’. Registered Dental Hygienist.

[5] Xiao J, Fiscella KA, Meyerowitz C (2021). mDentistry: A powerful tool to improve oral health of a broad population in the digital era. J Am Dent Assoc.

[6] Gilbert GH, Williams OD, Korelitz JJ, Fellows JL, Gordan VV, Makhija SK, Meyerowitz C, Oates TW, Rindal DB, Benjamin PL, Foy PJ, National Dental PBRN Collaborative Group (2013). Purpose, structure, and function of the United States National Dental Practice-Based Research Network. J Dent.

[7] Lopes JP, Dias TMR, Carvalho DBF, Oliveira JFD, Cavalcante RB, Oliveira VCD (2019). Evaluation of digital vaccine card in nursing practice in vaccination room. Rev Lat Am Enfermagem.

[8] Friesen EL (2017). Measuring AT usability with the modified System Usability Scale (SUS). Stud Health Technol Inform.

[9] Pillalamarri SS, Huyett LM, Abdel-Malek A (2018). Novel Bluetooth-enabled tubeless insulin pump: A user experience design approach for a connected digital diabetes management platform. J Diabetes Sci Technol.

[10] Sustersic M, Gauchet A, Kernou A, Gibert C, Foote A, Vermorel C, Bosson J (2018). A scale assessing doctor-patient communication in a context of acute conditions based on a systematic review. PLoS One.

[11] Irving M, Stewart R, Spallek H, Blinkhorn A (2017). Using teledentistry in clinical practice as an enabler to improve access to clinical care: A qualitative systematic review. J Telemed Telecare.

[12] Teoh J, Hsueh A, Mariño R, Manton D, Hallett K (2018). Economic evaluation of teledentistry in cleft lip and palate patients. Telemed J E Health.

[13] Joe J, Chaudhuri S, Le T, Thompson H, Demiris G (2015). The use of think-aloud and instant data analysis in evaluation research: Exemplar and lessons learned. J Biomed Inform.

[14] Keogh A, Dorn JF, Walsh L, Calvo F, Caulfield B (2020). Comparing the usability and acceptability of wearable sensors among older Irish adults in a real-world context: Observational study. JMIR Mhealth Uhealth.

[15] Zijp T, Touw D, van Boven J (2020). User acceptability and technical robustness evaluation of a novel smart pill bottle prototype designed to support medication adherence. PPA.

[16] Morse JM (2016). Determining sample size. Qual Health Res.

[17] Reeder B, Drake C, Ozkaynak M, Wald HL (2019). Usability testing of a mobile clinical decision support app for urinary tract infection diagnosis in nursing homes. J Gerontol Nurs.

[18] Simons D, De Bourdeaudhuij I, Clarys P, De Cocker K, Vandelanotte C, Deforche B (2018). A smartphone app to promote an active lifestyle in lower-educated working young adults: Development, usability, acceptability, and feasibility study. JMIR Mhealth Uhealth.

[19] Tobias G, Spanier AB (2020). Developing a mobile app (iGAM) to promote gingival health by professional monitoring of dental selfies: user-centered design approach. JMIR Mhealth Uhealth.

[20] Chen R, Santo K, Wong G, Sohn W, Spallek H, Chow C, Irving M (2021). Mobile apps for dental caries prevention: Systematic search and quality evaluation. JMIR Mhealth Uhealth.

[21] Xiao J, Luo J, Ly-Mapes O, Wu TT, Dye T, Al Jallad N, Hao P, Ruan J, Bullock S, Fiscella K (2021). Assess A Smartphone App (AICaries) that uses artificial intelligence to detect dental caries in children and provide interactive oral health education: Protocol for design and usability testing. JMIR Res Protoc.

[22] The National Dental Practice-Based Research Network.

