# Supplementary material for: Assessment of an Innovative Mobile Dentistry eHygiene Model Amid the COVID-19 Pandemic in the National Dental Practice–Based Research Network: Protocol for Design, Implementation, and Usability Testing
Source: JMIR Res Protoc. 2021 Oct 26;10(10):e32345. doi: 10.2196/32345 (PMC8549859; doi:10.2196/32345)
Supplement: Multimedia Appendix 2 [file resprot_v10i10e32345_app2.docx]

**Dentist CRF 1-1: Baseline questionnaire**

Instruction: Please answer the following questions regarding your thoughts on the **Standard Hygiene Exam Model** where you (dentist) will go into the hygiene room and conduct an in-person examination for your patient.

1. In the setting of COVID-19 outbreak, do you change PPEs when switching from seeing chair side patient to a hygiene check patient?

A. Yes, every time.

B. Yes, sometime

C. Never

2. How much money do you spend on purchasing a set of PPEs including disposable gowns, gloves, surgical masks?

A. Less than $5

B. $5-10

C. $10-20

D. I don’t know

3. Do you charge PPEs fees since the COVID-19 pandemic?

A. Yes, every patient

B. Yes, some patients

C. No

4. How challenging has purchasing PPE supply has been since the COVID-19 pandemic?

A. Not at all challenging

B. Somewhat challenging

C. Very challenging

5. How much time you generally spend on conducting a routine hygiene check?

A. < 5 minutes

B. 5-10 minutes

C. >10 minutes

D. I don’t conduct hygiene check during the hygiene appointment

6. Do you feel you have enough time to conduct routine hygiene checks?

A. Yes, all the time

B. Not always

C. No, I always run into time management issue while conducting hygiene checks

7. Do you feel your patients understand what you explain about their oral health and/or the treatment you recommend during hygiene check?

A. Yes, every patient

B. Yes, some patients

C. I am not sure

8. Do your patients follow through with the advice and treatment plan you recommend during the hygiene check?

A. Yes, every patient

B. Yes, some patients

C. No

D. I am not sure

**Electronic Device and Digital Image Use**

9. Do you have a smartphone?

A. Yes (Continue with Question 10)

B. No (Jump to Question 11)

10. Do you use medical care related Apps on your phone (e.g. Mychart, eClinicalWorks, SimplePractice)?

A. Yes

B. No

C. I am not sure what Apps you are referring to

11. Do you use dental care related Apps on your phone?

A. Yes (Please specify________)

B. No

C. I am not sure what Apps you are referring to

12. Does your office routinely take intraoral images for patients?

A. Yes, every patient

B. Yes, some patients

C. Never

13. Do you use images on the computer or software to conduct patient education and/or treatment planning?

A. Yes, every patient

B. Yes, some patients

C. Never

**Dentist CRF 2-1: eHygiene System Usability Scale (SUS)**

**Instruction:** For each of the following statements, mark one box that best describes your reactions to the eHygiene exam model. **In the eHygiene model**, the hygienist in your office will take a set of teeth photos for your patient during the regular hygiene visit. It might take 5-8 minutes. The dental office will then schedule a virtual visit between you (dentist) and your patient to review exam findings using these teeth photos. You (dentist) will also review treatment plan with your patient at the virtual visit. Depends on the complexity of your patient’s oral health. The virtual visit might take 10-30 minutes.

| No | Question description | Strongly disagree |  |  |  | Strongly agree | Score |
| --- | --- | --- | --- | --- | --- | --- | --- |
| 1 | I think that I would like to use the eHygiene exam model frequently. |  |  |  |  |  |  |
|  | *Scoring algorism (point)* | 0 | 1 | 2 | 3 | 4 |  |
| 2 | I found the eHygiene exam model unnecessarily complex. |  |  |  |  |  |  |
|  | *Scoring algorism (point)* | 4 | 3 | 2 | 1 | 0 |  |
| 3 | I thought the eHygiene exam model was easy to use. |  |  |  |  |  |  |
|  | *Scoring algorism (point)* | 0 | 1 | 2 | 3 | 4 |  |
| 4 | I think that I would need the support of a technical person (other than the hygienist) to be able to use the eHygiene exam model. |  |  |  |  |  |  |
|  | *Scoring algorism (point)* | 4 | 3 | 2 | 1 | 0 |  |
| 5 | I found the various steps in the eHygiene exam model were well integrated. |  |  |  |  |  |  |
|  | *Scoring algorism (point)* | 0 | 1 | 2 | 3 | 4 |  |
| 6 | I thought there was too much inconsistency in the eHygiene exam model. |  |  |  |  |  |  |
|  | *Scoring algorism (point)* | 4 | 3 | 2 | 1 | 0 |  |
| 7 | I would imagine that most people would learn to use the eHygiene exam model very quickly. |  |  |  |  |  |  |
|  | *Scoring algorism (point)* | 0 | 1 | 2 | 3 | 4 |  |
| 8 | I found the eHygiene exam model very awkward to use. |  |  |  |  |  |  |
|  | *Scoring algorism (point)* | 4 | 3 | 2 | 1 | 0 |  |
| 9 | I felt very confident using eHygiene exam model. |  |  |  |  |  |  |
|  | *Scoring algorism (point)* | 0 | 1 | 2 | 3 | 4 |  |
| 10 | I needed to learn a lot of things before I could start with the eHygiene exam model. |  |  |  |  |  |  |
|  | *Scoring algorism (point)* | 4 | 3 | 2 | 1 | 0 |  |

***Total score = sum of all 10 items * 2.5**

**Dentist CRF 2-2: Post eHygiene questionnaire**

Instruction: Please answer the following questions regarding your thoughts on the **eHygiene Exam Model**.

1. How much time did you spend initiating the eHygiene virtual visit? Initiation includes logging into the software interface and starting video call with your patient.

Please input your answer: _____________ minutes

2. How much time did you spend conducting the eHygiene virtual visit?

Please input your answer: _____________ minutes

3. When did you conduct eHygiene virtual visit? (Choose all that apply)

A. During the day between treating patients

B. Lunch break

C. Evening when I finished daily work

D. Other (Please detail______)

4. Do you feel you will add to your working time if you conduct eHygiene virtual visits?

A. Yes, all the time

B. Sometimes

C. No

D. I am not sure

5. Which types of patients do you think should be considered for eHygiene virtual visits? (Choose all that apply)

A. None

B. Patients with good oral health who had no restorative/periodontal treatment in the past 1+ year

C. Patients with non-urgent oral diseases (caries, periodontal pocket deeper than 4mm, etc.) identified by hygienists during cleaning.

D. Patients with oral mucosal lesions identified by hygienists during cleaning.

E. All patients

6. Do you feel your patient understood what you explained about her/his oral health and/or the treatment you recommend during eHygiene visit?

A. Yes

B. No

C. I am not sure

7. Do you believe your patient will follow through with the advice and treatment plan you recommended during the eHygiene visit?

A. Yes

B. No

C. I am not sure

8. Do you have any suggestions to improve eHygiene exam model?

A. Yes (Please explain in detail___________________)

B. No

9. Have you completed the eHygiene study virtual visit with this patient?

A. Yes (This answer will trigger sending an email to patient for patient evaluation)

B. No
