# Supplementary material for: Assessment of an Innovative Mobile Dentistry eHygiene Model Amid the COVID-19 Pandemic in the National Dental Practice–Based Research Network: Protocol for Design, Implementation, and Usability Testing
Source: JMIR Res Protoc. 2021 Oct 26;10(10):e32345. doi: 10.2196/32345 (PMC8549859; doi:10.2196/32345)
Supplement: Multimedia Appendix 3 [file resprot_v10i10e32345_app3.docx]

**Hygienist CRF 1-1: Baseline questionnaire**

Instruction: Please answer the following questions regarding your thoughts on the **Standard Hygiene Exam Model** where dentists will come into your room and conduct an in-person examination for your patient.

1. How much time does the dentist in your office spend on conducting a routine hygiene check?

A. < 5 minutes

B. 5-10 minutes

C. >10 minutes

D. The dentist in my office does not conduct routine hygiene check during the hygiene appointment

2. How much time do you usually wait for the dentist in your office to conduct a routine hygiene check for your patient?

A. < 5 minutes

B. 5-10 minutes

C. >10 minutes

D. The dentist in my office does not conduct hygiene check during the hygiene appointment

3. What percentage of your patient refuse a routine hygiene check exam?

A. <25%

B. 26-50%

C. 51-75%

D. >75%

E. I don’t know

4. What are the common reasons that your patients would request a waiver for a hygiene check? (Choose all that apply)

A. Waiting time

B. Cost

C. Do not think it’s needed

D. I don’t know

5. Do you feel your patients understand what the dentist explains about their oral health and/or the treatment the dentist recommends during hygiene check?

A. Yes, every patient

B. Yes, some patients

C. No

D. I am not sure

6. Does your patient follow through with the advice and treatment plan recommended by the dentist during the hygiene check?

A. Yes, every patient

B. Yes, some patients

C. No

D. I am not sure

**Electronic Device Use**

7. Do you have a smartphone?

A. Yes (Continue with Question 8)

B. No (Jump to Question 9)

8. Do you use medical care related Apps on your phone (e.g. Mychart, eClinicalWorks, SimplePractice)?

A. Yes

B. No

C. I am not sure what Apps you are referring to

9. Do you use dental care related Apps on your phone?

A. Yes (Please specify________)

B. No

C. I am not sure what Apps you are referring to

10. Do you routinely take intraoral images for your patients in your office?

A. Yes, every patient

B. Yes, some patients

C. Never

11. Do you use images on the computer or software to conduct patient education and/or treatment planning?

A. Yes, every patient

B. Yes, some patients

C. Never

**Hygienist CRF 2-1: eHygiene System Usability Scale (SUS)**

**Instruction:** For each of the following statements, mark one box that best describes your reactions to the eHygiene exam model. **In the eHygiene model**, you (hygienist) will take a set of teeth photos for your patient during the regular hygiene visit. It might take 5-8 minutes. The dental office will then schedule a virtual visit between the dentist and your patient to review exam findings using these teeth photos. The dentist will also review treatment plan with your patient at the virtual visit. Depends on the complexity of your patient’s oral health. The virtual visit might take 10-30 minutes.

| No | Question description | Strongly disagree |  |  |  | Strongly agree | Score |
| --- | --- | --- | --- | --- | --- | --- | --- |
| 1 | I think that I would like to use the eHygiene exam model frequently. |  |  |  |  |  |  |
|  | *Scoring algorism (point)* | 0 | 1 | 2 | 3 | 4 |  |
| 2 | I found the eHygiene exam model unnecessarily complex. |  |  |  |  |  |  |
|  | *Scoring algorism (point)* | 4 | 3 | 2 | 1 | 0 |  |
| 3 | I thought the eHygiene exam model was easy to use. |  |  |  |  |  |  |
|  | *Scoring algorism (point)* | 0 | 1 | 2 | 3 | 4 |  |
| 4 | I think that I would need the support of a technical person to be able to use the eHygiene exam model. |  |  |  |  |  |  |
|  | *Scoring algorism (point)* | 4 | 3 | 2 | 1 | 0 |  |
| 5 | I found the various steps in the eHygiene exam model were well integrated. |  |  |  |  |  |  |
|  | *Scoring algorism (point)* | 0 | 1 | 2 | 3 | 4 |  |
| 6 | I thought there was too much inconsistency in the eHygiene exam model. |  |  |  |  |  |  |
|  | *Scoring algorism (point)* | 4 | 3 | 2 | 1 | 0 |  |
| 7 | I would imagine that most people would learn to use the eHygiene exam model very quickly. |  |  |  |  |  |  |
|  | *Scoring algorism (point)* | 0 | 1 | 2 | 3 | 4 |  |
| 8 | I found the eHygiene exam model very awkward to use. |  |  |  |  |  |  |
|  | *Scoring algorism (point)* | 4 | 3 | 2 | 1 | 0 |  |
| 9 | I felt very confident using eHygiene exam model. |  |  |  |  |  |  |
|  | *Scoring algorism (point)* | 0 | 1 | 2 | 3 | 4 |  |
| 10 | I needed to learn a lot of things before I could start with the eHygiene exam model. |  |  |  |  |  |  |
|  | *Scoring algorism (point)* | 4 | 3 | 2 | 1 | 0 |  |

***Total score = sum of all 10 items * 2.5**

**Hygienist CRF 2-2: Post eHygiene questionnaire**

Instruction: Please answer the following questions regarding your thoughts on the **eHygiene Exam Model**.

1. How much time did you spend setting up the device and software before you start taking intraoral photos during the eHygiene visit?

Please input your answer: _____________ minutes

2. How much time did you spend taking intraoral photos during eHygiene virtual visit?

Please input your answer: _____________ minutes

3. Did your patient experience any discomfort when you took intraoral photos?

A. Yes

B. No

4. When did you take intraoral photos during the eHygiene visit? (Choose all that apply)

A. Before I started the prophy

B. After I completed the prophy

5. Do you feel you will add to your working time if you conduct eHygiene virtual visits?

A. Yes, all the time

B. Depends on the day

C. No

D. I am not sure

6. Which types of patients do you think should be considered for eHygiene virtual visits? (choose all that apply)

A. None

B. Patients with good oral health who had no restorative/periodontal treatment in the past 1+ year

C. Patients with non-urgent oral diseases (caries, periodontal pocket deeper than 4mm, etc.) identified by hygienists during cleaning.

D. Patients with oral mucosal lesions identified by hygienists during cleaning.

E. All patients

7. Have you completed the study visit with this patient, including taking intraoral photos and scheduling a virtual visit for patient and dentist?

A. Yes (This answer will trigger hygienist payment readiness, and send an email to patient and dentist with link to TeleDent and scheduled date/time for the virtual visit)

B. No
