# Supplementary material for: Assessment of an Innovative Mobile Dentistry eHygiene Model Amid the COVID-19 Pandemic in the National Dental Practice–Based Research Network: Protocol for Design, Implementation, and Usability Testing
Source: JMIR Res Protoc. 2021 Oct 26;10(10):e32345. doi: 10.2196/32345 (PMC8549859; doi:10.2196/32345)
Supplement: Multimedia Appendix 4 [file resprot_v10i10e32345_app4.docx]

**Patient CRF 1-1: Baseline questionnaire**

Instruction: Please answer the following questions regarding your thoughts on the **Standard Hygiene Exam** where dentists will come into the dental room conduct an in-person examination during your cleaning appointment.

1. How much time does the dentist spend on conducting a routine hygiene check for you?

A. < 5 minutes

B. 5-10 minutes

C. >10 minutes

D. The dentist in my office does not conduct hygiene check during the hygiene appointment

2. How much time do you usually wait for the dentist in your office to conduct a routine hygiene check?

A. < 5 minutes

B. 5-10 minutes

C. >10 minutes

D. The dentist in my office does not conduct hygiene check during the hygiene appointment

3. Have you ever left the dental office visit without waiting for the dentist to perform the hygiene check?

A. Yes (Continue with Question 4)

B. No (Jump to Question 5)

C. I don’t remember

4. What are the reasons you requested to leave the office without the dentist doing an exam after a hygiene visit? (Choose all that apply)

A. Waiting time is too long

B. Cost of the exam

C. I do not think it’s needed

D. The office told me I don’t need it

5. Do you follow through with the advice and treatment plan recommended by the dentist recommend during the hygiene check?

A. Yes, all the time

B. Yes, some time

C. No, I never understand

**Electronic Device Use**

6. Do you have a smartphone?

A. Yes (Continue with Question 7)

B. No (Jump to Question 9)

7. Do you use medical care related Apps on your phone (e.g. Mychart, eClinicalWorks, SimplePractice)?

A. Yes

B. No

8. Do you use dental care related Apps on your phone?

A. Yes (Please specify________)

B. No

C. I am not sure what Apps you are referring to

9. Have you tried to use a cell phone to take photos of your teeth or mouth?

A. Yes, many times

B. Yes, a few times

C. Never

10. Do you think it will be helpful if the dental professionals use images on the computer or software to explain your oral health?

A. Yes, very helpful

B. Yes, to some degree

C. No, not helpful

D. I don’t know

**Dentist-Patient Communication perceived by patients (DPC)**

| No | Questions | No | Possibly no | Possibly yes | Yes |
| --- | --- | --- | --- | --- | --- |
| 1 | Did your dentist listen to you carefully during the standard hygiene exam visit? |  |  |  |  |
| 2 | Did your dentist allow you to talk without interrupting you during the standard hygiene exam visit? |  |  |  |  |
| 3 | Did your dentist encourage you to express yourself /talk during the standard hygiene exam visit? |  |  |  |  |
| 4 | Did your dentist explain the findings from the intraoral photos and/or x-rays thoroughly? |  |  |  |  |
| 5 | Do you feel that the dentist understood you? |  |  |  |  |
| 6 | Was it easy to understand what the doctor said during the standard hygiene exam visit? |  |  |  |  |
| 7 | Do you feel you were given all the necessary information during the standard hygiene exam visit? |  |  |  |  |
| 8 | Did the dentist explain the advantages and disadvantages of the treatment or care strategy during the standard hygiene exam visit? |  |  |  |  |
| 9 | Did the dentist involve you in the decision-making during the standard hygiene exam visit? |  |  |  |  |
| 10 | In your opinion, did your dentist have a reassuring attitude and way of talking during the standard hygiene exam visit? |  |  |  |  |
| 11 | Do you think your dentist was in general respectful during the standard hygiene exam visit? |  |  |  |  |
| 12 | Did your dentist make sure that you understood his/her explanations and instructions during the standard hygiene exam visit? |  |  |  |  |
| 13 | Do you think your dentist told the whole truth? |  |  |  |  |
| 14 | Do you have confidence in your dentist after the during the standard hygiene exam visit? |  |  |  |  |
| 15 | Did your dentist reply to all your expectations and concerns? |  |  |  |  |

*Total score of DPC: 60*

*Scoring algorism: No (1 point), Possibly no (2 point), Possibly yes (3 point), Yes (4 point)*

**Patient CRF 2-1: eHygiene System Usability Scale (SUS)**

**Instruction:** For each of the following statements, mark one box that best describes your reactions to the **eHygiene exam**. In the eHygiene model, the hygienist will take a set of teeth photos for you during your regular hygiene visit. It might take 5-8 minutes. The dental office will then schedule a virtual visit between you and your dentist to review exam findings using these teeth photos. You dentist will also review treatment plan with you at the virtual visit. Depends on the complexity of your oral health. The virtual visit might take 10-30 minutes.

| No | Question description | Strongly disagree |  |  |  | Strongly agree | Score |
| --- | --- | --- | --- | --- | --- | --- | --- |
| 1 | I think that I would like to use the eHygiene exam frequently. |  |  |  |  |  |  |
|  | *Scoring algorism (point)* | 0 | 1 | 2 | 3 | 4 |  |
| 2 | I found the eHygiene exam unnecessarily complex. |  |  |  |  |  |  |
|  | *Scoring algorism (point)* | 4 | 3 | 2 | 1 | 0 |  |
| 3 | I thought the eHygiene exam was easy to use. |  |  |  |  |  |  |
|  | *Scoring algorism (point)* | 0 | 1 | 2 | 3 | 4 |  |
| 4 | I think that I would need the support of a technical person (other than my dentist and hygienist) to be able to use the eHygiene exam. |  |  |  |  |  |  |
|  | *Scoring algorism (point)* | 4 | 3 | 2 | 1 | 0 |  |
| 5 | I found the various steps in the eHygiene exam were well integrated. |  |  |  |  |  |  |
|  | *Scoring algorism (point)* | 0 | 1 | 2 | 3 | 4 |  |
| 6 | I thought there was too much inconsistency in the eHygiene exam. |  |  |  |  |  |  |
|  | *Scoring algorism (point)* | 4 | 3 | 2 | 1 | 0 |  |
| 7 | I would imagine that most people would learn to use the eHygiene exam very quickly. |  |  |  |  |  |  |
|  | *Scoring algorism (point)* | 0 | 1 | 2 | 3 | 4 |  |
| 8 | I found the eHygiene exam very awkward to use. |  |  |  |  |  |  |
|  | *Scoring algorism (point)* | 4 | 3 | 2 | 1 | 0 |  |
| 9 | I felt very confident using eHygiene exam. |  |  |  |  |  |  |
|  | *Scoring algorism (point)* | 0 | 1 | 2 | 3 | 4 |  |
| 10 | I needed to learn a lot of things before I could start with the eHygiene exam. |  |  |  |  |  |  |
|  | *Scoring algorism (point)* | 4 | 3 | 2 | 1 | 0 |  |

***Total score = sum of all 10 items * 2.5**

**Patient CRF 2-2: Post eHygiene questionnaire**

Instruction: Please answer the following questions regarding your thoughts on the **eHygiene Exam**.

**eHygiene Exam**

1. Did you feel comfortable when the hygienist took intraoral photos for you?

A. Very comfortable

B. Somewhat comfortable

C. Somewhat uncomfortable

D. Extremely uncomfortable

2. Did the eHygiene virtual visit occur during your work hours?

A. Yes

B. No

C. No, I am not employed at this time.

3. How much time did you spend logging into software for the eHygiene virtual visit with the dentist?

Please input your answer (approximately): _____________ minutes

4. Do you feel you had enough time to communicate with the dentist during eHygiene virtual visit?

A. Yes, plenty of time

B. No, I wish it would have been longer

**Dentist-Patient Communication Perceived by Patients (DPC)**

| No | Questions | No | Possibly no | Possibly yes | Yes |
| --- | --- | --- | --- | --- | --- |
| 1 | Did your dentist listen to you carefully during the eHygiene virtual visit? |  |  |  |  |
| 2 | Did your dentist allow you to talk without interrupting you during the eHygiene virtual visit? |  |  |  |  |
| 3 | Did your dentist encourage you to express yourself /talk during the eHygiene virtual visit? |  |  |  |  |
| 4 | Did your dentist explain the findings from the intraoral photos and/or x-rays thoroughly? |  |  |  |  |
| 5 | Do you feel that the dentist understood you? |  |  |  |  |
| 6 | Was it easy to understand what the doctor said during the eHygiene virtual visit? |  |  |  |  |
| 7 | Do you feel you were given all the necessary information during the eHygiene virtual visit? |  |  |  |  |
| 8 | Did the dentist explain the advantages and disadvantages of the treatment or care strategy during the eHygiene virtual visit? |  |  |  |  |
| 9 | Did the dentist involve you in the decision-making during the eHygiene virtual visit? |  |  |  |  |
| 10 | In your opinion, did your dentist have a reassuring attitude and way of talking during the eHygiene virtual visit? |  |  |  |  |
| 11 | Do you think your dentist was in general respectful during the eHygiene virtual visit? |  |  |  |  |
| 12 | Did your dentist make sure that you understood his/her explanations and instructions during the eHygiene virtual visit? |  |  |  |  |
| 13 | Do you think your dentist told the whole truth? |  |  |  |  |
| 14 | Do you have confidence in your dentist after the during the eHygiene virtual visit? |  |  |  |  |
| 15 | Did your dentist reply to all your expectations and concerns? |  |  |  |  |

*Total score of DPC: 60*

*Scoring algorism: No (1 point), Possibly no (2 point), Possibly yes (3 point), Yes (4 point)*
