# Supplementary material for: Assessment of an Innovative Mobile Dentistry eHygiene Model Amid the COVID-19 Pandemic in the National Dental Practice–Based Research Network: Protocol for Design, Implementation, and Usability Testing
Source: JMIR Res Protoc. 2021 Oct 26;10(10):e32345. doi: 10.2196/32345 (PMC8549859; doi:10.2196/32345)
Supplement: Multimedia Appendix 5 [file resprot_v10i10e32345_app5.docx]

**eHygiene Interview guide**

**(Semi-structured)**

**Notes:**

- This is a semi-structured interview guide designed to be conducted with patients, dentists and hygienists in a 30-min phone-interview setting.
- This interview includes questions to assess user’s perception of eHygiene exam model.
- The phone interview will be conducted by research assistant (a dentist) trained by Dr. Kevin Fiscella, MD, MPH (co-investigator) who has expertise in mhealth and qualitative research.

***Date: _____/_____/_____ (mm/dd/yy)***

***Interviewer: _____________________***

***Study Participant number: __________***

***Study participant role:***  Dentist  Hygienist  Patient

***Introduction (script)***

*“Hello, my name is ______. I am with the eHygiene study group and I would like to talk with you about your experience of using eHygiene virtual exam.”*

*“Thank you for agreeing to speak to me! This interview will take approximately 30 minutes and will be recorded.”*

*“What we talked about in this interview will not be released to personnel outside of our study team”*

*“Your participation is completely voluntary. If you do not wish to answer any question you do not have to do so. You are free to end the conversation at any time”*

*“Before I turn on the tape recorder do you have any questions?”*

*“Now I am going to turn on the tape recorder, is that ok?”*

*“For the questions I am about to ask you are related to eHygiene exam model. The eHygiene exam model refers to the study you participated, where the hygienists in dental office took a set of teeth photos during youre regular hygiene visit. The dentists then conducted a virtual visit with you to review exam findings using these teeth photos and discussed your treatment plan.”*

**Dentist and Hygienist portion**

**Perception**

1. Can you talk about your overall experience of using eHygiene virtual exam?
2. Please tell us any challenges or frustrations you encountered while using eHygiene virtual exam.

*Prompts – What kind of challenges / frustrations*

*Did you resolve it?*

*How did you resolve it?*

*Are there other challenges? Any more (until no more challenges)*

1. What are the benefits of eHygiene virtual exam?

*Prompts – Benefits to dental practice?*

*Benefits to patients?*

*Any other benefits (until no more)?*

1. What are the drawbacks of eHygiene virtual exam?

*Prompts – Patient communication*

*Workflow*

*Technical problem*

*Anything else (until no more)?*

5. What do you see the role of eHygiene virtual exam in your practice, if any?

*Prompts – If yes, or no, could you please tell me why?*

**Service strengthening**

1. What would you suggest to improve eHygiene virtual exams?
2. What support do you need to feel comfortable using eHygiene virtual exams?
3. Are there any system changes that you think might be needed to improve utilization of the eHygiene virtual exam model?

*Prompts – Insurance reimbursement*

*Use mass media or social media*

**Smart phone application use**

1. What is your view on using smart phone applications to deliver oral health education to patients?
2. What suggestion do you have for using smart phones to manage patient oral health?

**Conclusion**

*“What other things you would like us to know about your experience or thoughts about eHygiene virtual exams?”*

“Thank you!”

**Patient portion**

**Perception**

1. Can you talk about your overall experience of using eHygiene virtual exam?
2. Can you talk about challenges or frustrations you encountered while using eHygiene virtual exam?

*Prompts – What kind of challenges / frustrations*

*Did you resolve it?*

*How did you resolve it?*

*Anything else (until no more)?*

1. What are the benefits of using eHygiene virtual exam?

*Prompts – Safety?*

*Convenience?*

*Communication time with dentist?*

*Any other benefits (until no more)?*

1. How much do you trust the eHygiene exam model is sufficient for your dentist to obtain information about your oral health as a regular checkup?
2. What else would you wish to include in the current eHygiene exam model?
3. How likely would you recommend your family members and friends to use eHygiene exam?

**Service strengthening**

1. What changes will you suggest to improve eHygiene virtual dental exams?

*Prompt – do you have suggestions for addressing challenges you mentioned earlier.*

**Smart phone application use**

1. What kind of oral health related smartphone app would you like to use?
2. If there is a smartphone app that helps you take photo of your teeth, and you can check with your dentist whether there are problems, will you use it?

*Prompt – Why (Why not)?*

*Do you think that will help maintain or improve your oral health?*

**Patients taking teeth photos (SELFIE session)** – This domain is for patients who conducted SELFIE session.

1. What was your experience in taking pictures of your teeth?

2. What problems did you run into?

*Prompt – Any problems during connecting intraoral cameras with tablet, using photo-taking module in the Teledent software, taking photos, storing photos in the Teledent?.*

*Other problems (until no more)?*

3. How did you resolve these problems?

4. What suggestions do you have for making it easier for patients take pictures of their teeth?

5. If sending pictures of your teeth to your dentist made your visit shorter, would it be worth your time and effort?

*Prompt – Why (Why not)?*

**Conclusion**

*“What other things you would like us to know about your experience or thoughts about eHygiene virtual exams?”*

“Thank you!”
