# Supplementary material for: Assessment of an Innovative Mobile Dentistry eHygiene Model Amid the COVID-19 Pandemic in the National Dental Practice–Based Research Network: Protocol for Design, Implementation, and Usability Testing
Source: JMIR Res Protoc. 2021 Oct 26;10(10):e32345. doi: 10.2196/32345 (PMC8549859; doi:10.2196/32345)
Supplement: Multimedia Appendix 6 [file resprot_v10i10e32345_app6.docx]

**SELFIE Intraoral Photos Assessment Sheet**

**(Study Team Use)**

Note: The quantity and quality of intraoral photos will be assessed by a study research assistant (a dentist) trained by study PIs.

| Roles  Types of images | Hygienist taken photos | | | | Patient taken photos | | |
| --- | --- | --- | --- | --- | --- | --- | --- |
|  | Total | Clear images | Diagnostic images | Total | | Clear images | Diagnostic images |
| Number of total intraoral photos |  |  |  |  | |  |  |
| **Breakdown** | | | | | | | |
| Front view |  |  |  |  | |  |  |
| Buccal-Upper molars (left) |  |  |  |  | |  |  |
| Buccal-Upper molars (right) |  |  |  |  | |  |  |
| Lingual-Upper molars (left) |  |  |  |  | |  |  |
| Lingual-Upper molars (right) |  |  |  |  | |  |  |
| Lingual- upper premolars (left) |  |  |  |  | |  |  |
| Lingual- Upper premolars (right) |  |  |  |  | |  |  |
| Lingual-Upper front |  |  |  |  | |  |  |
| Lingual-Lower molars (left) |  |  |  |  | |  |  |
| Lingual-Lower molars (right) |  |  |  |  | |  |  |
| Lingual-Lower premolars (left) |  |  |  |  | |  |  |
| Lingual-Lower premolars (right) |  |  |  |  | |  |  |
| Lingual-Lower front |  |  |  |  | |  |  |
| Occlusal-Upper molars (left) |  |  |  |  | |  |  |
| Occlusal-Upper molars (right) |  |  |  |  | |  |  |
| Occlusal- upper premolars (left) |  |  |  |  | |  |  |
| Occlusal- Upper premolars (right) |  |  |  |  | |  |  |
| Occlusal-Upper front |  |  |  |  | |  |  |
| Occlusal-Lower molars (left) |  |  |  |  | |  |  |
| Occlusal-Lower molars (right) |  |  |  |  | |  |  |
| Occlusal-Lower premolars (left) |  |  |  |  | |  |  |
| Occlusal-Lower premolars (right) |  |  |  |  | |  |  |
| Occlusal-Lower front |  |  |  |  | |  |  |
