# Supplementary material for: Assessment of an Innovative Mobile Dentistry eHygiene Model Amid the COVID-19 Pandemic in the National Dental Practice–Based Research Network: Protocol for Design, Implementation, and Usability Testing
Source: JMIR Res Protoc. 2021 Oct 26;10(10):e32345. doi: 10.2196/32345 (PMC8549859; doi:10.2196/32345)
Supplement: Multimedia Appendix 7 [file resprot_v10i10e32345_app7.docx]

**SELFIE Patient Taking Photos Qualitative Assessment Sheet**

**(Study Team Use)**

Notes:

- In the SELFIE session, patients will take a series of intraoral photos with remote guidance of a study hygienist. The session will be recorded.
- The SELFIE video assessment will be conducted by research assistant (a dentist) trained (by Dr. Kevin Fiscella, MD, MPH (co-investigator) who has expertise in mHealth and qualitative research).

| **Tasks by the patients** | **Time-spent (minute)** | **Challenges** | | | |
| --- | --- | --- | --- | --- | --- |
|  |  | **No challenges** | **Cosmetic (minor)** | **Severe** | **Critical** |
| Connecting intraoral cameras with tablet |  |  |  |  |  |
| Locate photo-taking module in Teledent software |  |  |  |  |  |
| Using cheek retractor |  |  |  |  |  |
| Taking front-view teeth photos |  |  |  |  |  |
| Taking posterior teeth photos |  |  |  |  |  |
| Ensure intra-oral photos is stored in Teledent |  |  |  |  |  |

Note:

*Cosmetic*: minor

*Severe*: major delay and/or frustration

*Critical*: requiring assistance to proceed
